# Shot noise-mitigated secondary electron imaging with ion count-aided microscopy

**Published:** 2024-07-08

**Authors:** Akshay Agarwal, Leila Kasaei, Xinglin He, Ruangrawee Kitichotkul, Oğuz Kağan Hitit, Minxu Peng, J. Albert Schultz, Leonard C. Feldman, Vivek K Goyal

**Affiliations:** 1 Department of Electrical and Computer Engineering, Boston University; 2 Department of Physics, Rutgers University; 3 Ionwerks Inc.

## Abstract

Modern science is dependent on imaging on the nanoscale, often achieved through processes that detect secondary electrons created by a highly focused incident charged particle beam. Multiple types of measurement noise limit the ultimate trade-off between the image quality and the incident particle dose, which can preclude useful imaging of dose-sensitive samples. Existing methods to improve image quality do not fundamentally mitigate the noise sources. Furthermore, barriers to assigning a physically meaningful scale make the images qualitative. Here we introduce ion count-aided microscopy (ICAM), which is a quantitative imaging technique that uses statistically principled estimation of the secondary electron yield. With a readily implemented change in data collection, ICAM substantially reduces source shot noise. In helium ion microscopy, we demonstrate 3× dose reduction and a good match between these empirical results and theoretical performance predictions. ICAM facilitates imaging of fragile samples and may make imaging with heavier particles more attractive.

## Introduction

Secondary electron imaging (SEI) modalities such as scanning electron microscopy (SEM) and helium ion microscopy (HIM) are widely used in nanoscale characterization and analysis [1, 2]. SEM is employed in diverse applications such as critical-dimension metrology and inspection for semiconductor devices [3], materials characterization in geology [4], and examination of biological samples [5]. With its applicability to non-conducting materials (not requiring sample coating before imaging), HIM is especially useful in the high-resolution imaging of biological samples such as animal organs [6, 7], tumor cells [8], and viruses [9, 10]. In SEI, a beam of charged particles (electrons or ions) is raster scanned across the sample being imaged. At each scan location, the incident beam is held in place while it excites secondary electrons (SEs) that are detected by a secondary electron detector (SED). A detected signal intensity is converted to a pixel brightness value. Scanning over a rectangular grid forms the final image of the sample. Ideally, an SE image would be a map of the sample’s SE yield η, *i.e.*, the mean number of SEs generated per incident particle. However, since SEDs do not have sufficient energy resolution to count SEs, and the gain and efficiency of the detector are generally unknown to the user, conventional SE images are merely qualitative—they cannot be directly mapped to pixelwise SE yields.

The quality of SEI is affected by three sources of noise: random variation in the number of incident particles (*source shot noise*), in the number of emitted SEs for each incident particle (*target shot noise*), and in the signal produced by the SED in response to SEs (*detector noise*). These noise sources limit the image quality achievable in SEI at any given imaging dose. For rugged materials such as gold or copper, the image quality can be improved by increasing dose. However, for fragile, radiation-sensitive materials, increase in dose also increases the damage imparted during imaging, such as atomic displacement and radiolysis [11, 12]. Displacement damage (such as sputtering and defect creation) is especially significant for HIM as compared to SEM, and it is worse yet with gallium or neon beams [13].

One approach to reducing the impact of detector noise is to count pulses in the SED voltage signal to infer the number of SEs. Such counting has been accomplished by external pulse counting circuits [14] and in software [15, 16]. While this technique improves imaging signal-to-noise-ratio at low SE yields, it is ineffective at the higher yields achievable with ions [2], where multiple SEs may be detected simultaneously. Post-processing methods to reduce noise, such as Gaussian/Poisson deconvolution [17, 18], compressed sensing [19, 20, 21], inpainting [22], and adaptive scanning [23], rely on assumptions about the underlying image structure rather than on the statistics of the particle source or SE emission. Where applicable, these types of post-processing can be combined with more fundamental physics-based improvements.

In this work, we introduce ion count-aided microscopy (ICAM), which uses novel data processing to nearly eliminate source shot noise. The target shot noise is inherent to the measured beam–sample interactions, and the detector noise can be reduced by altering the choice of detector technology; thus, ICAM approaches the accuracy limits of SEI. We demonstrate ICAM with a helium ion microscope to achieve quantitative, nanoscale SE yield metrology. Building upon a time-resolved measurement concept developed under an assumption of perfect SE observation [24, 25, 26], we use the full waveform of the SED signal to infer ion incidence events. Using the number of observed ion incidence events improves SE yield estimation significantly; we demonstrate reduction of the dose required for a given image quality by up to a factor of 3. Theoretical analysis suggests that the dose reduction factor provided by ICAM is approximately equal to the SE yield η and thus can be much larger for other incident particles and samples [27]. Although specialized for SEI, our work demonstrates improvements in imaging that can be accomplished with careful consideration of the statistical properties of the underlying data. It may inspire similar considerations in other types of imaging and materials characterization.

## Results

### Data collection to allow incident ion counting

**Figure 1:**
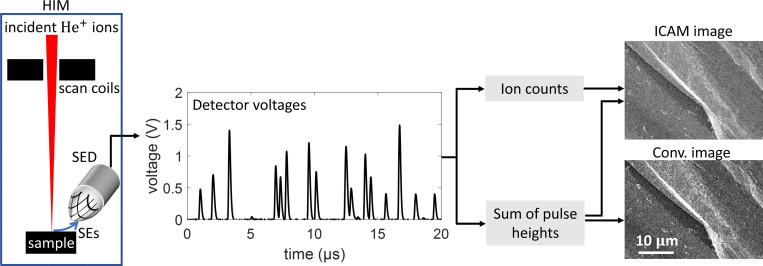
Schematic for ion count-aided microscopy in secondary electron imaging. The signal from the secondary electron detector on a helium ion microscope was outcoupled onto a digitizer for analysis. The heights ${\tilde{U}}_{i}$ and number $\tilde{M}$ of the SE voltage pulses in the detector signal were used to create both conventional and ICAM images. ICAM images consistently showed lower noise than conventional images.

Figure 1 shows a schematic of our imaging setup. We used a Zeiss Orion Plus helium ion microscope operated at 30 keV beam energy in our experiments. We outcoupled both the SED and the beam scan signals into a 12-bit, 100 MHz digitizer (Gage RazorExpress 1642), which sampled both signals at 10 ns. Figure 1 shows a 20μs snapshot of the SED voltage signal. The SED is typically an Everhart–Thornley detector [28], which consists of a scintillator followed by a photomultiplier. The signal generated by the SED consists of a series of voltage pulses of varying heights, where each pulse corresponds to a burst of detected SEs [29]. The mean full width at half maximum (FWHM) of the pulses is ∼160 ns [30]. In our experiments we used beam current $I_{b}=0.11\;\text{pA}$ (measured with a picoammeter connected to a Faraday cup), which was low enough to make pulse pile-up effects manageable.

To create images, we collected the SED voltage and beam scan signals at pixel dwell times $t_{d}$ varying between 2μs and 32μs for a 512 × 512-pixel image. These settings correspond to an incident dose $\lambda =I_{b}t_{d}/e$ between 1.4 and 22 ions per pixel, where e is the elementary charge. The maximum image size and dwell time were set by the available memory of the digitizer. Next, using custom MATLAB scripts, we extracted the SED voltage pulse heights ${\tilde{U}}_{i}$ and the number of pulses $\tilde{M}$ for each pixel. Neglecting pulse pile-up, $\tilde{M}$ would be the number of incident ions that produced one or more detected SEs. To account for pile-up, we divide the raw value of $\tilde{M}$ by a correction factor that accounts for the probability of pulse overlap to yield a value ${\tilde{M}}_{\text{corr}}$ [see Materials and Methods].

### Secondary electron yield estimation

We used the measurements $\left(\tilde{M},{\tilde{U}}_{1},{\tilde{U}}_{2},\ldots ,{\tilde{U}}_{\tilde{M}}\right)$ to create two pixelwise maps of η: conventional, which models typical SEI; and *ion count-aided* (ICA), which implements our source shot noise-mitigated estimator. While typical SEI uses only $V=\sum_{i}{\tilde{U}}_{i}$, the performance of ICAM demonstrates the value of using $\tilde{M}$ as well.

From its definition as the number of SEs generated per incident particle, it is natural for an estimate of SE yield η to be framed as

(1) $$\hat{\eta }=\frac{\text{estimated number of SEs}}{\text{estimated number of incident particles}}.$$

In bulk estimates of SE yield, measurements of the beam and sample currents may be used to calculate the numerator and denominator of this expression [31]. Our nanoscale mapping of η uses this intuitive expression with numerator and denominator estimated from $\left(\tilde{M},{\tilde{U}}_{1},{\tilde{U}}_{2},\ldots ,{\tilde{U}}_{\tilde{M}}\right)$.

#### Estimating number of SEs.

If we knew the mean voltage $c_{\mu }$ produced by the detector in response to one SE, $\hat{Y}=V/c_{\mu }$ would be an unbiased estimator for the number of SEs. As detailed in Materials and Methods and in figs. S1, S2, and S3 in the Supplementary Materials, we found that a linear probabilistic model accurately describes the statistics of the SE pulses. In this model, the number of incident particles and the number of emitted SEs both follow Poisson distributions, and the response of the detector to one SE follows a Gaussian distribution. By fitting this model to the pulse height distributions from samples with different η, we computed $c_{\mu }=0.163\;\text{V}$ for the microscope imaging settings used. We also computed the standard deviation of the detector’s voltage response to one SE, $c_{\sigma }=0.097\;\text{V}$. We used the estimate $\hat{Y}$ in the numerator of eq. (1) for both the conventional and ICA estimators.

#### Estimating number of incident particles.

Conventionally, any estimate of the number of incident particles M could depend only on the dose per pixel λ, since there is no effort to count incidence events. In that case, since the model specifies M∼Poisson(λ), the value of λ itself is the best estimate of M. In our method, we estimate M using the count of ions that produced at least one SE, $\tilde{M}$ corrected for pulse-pileup effects. Under our standard statistical assumptions, $\text{E}[M|\tilde{M}]=\tilde{M}+\text{λ}e^{-\eta }$. The $\text{λ}e^{-\eta }$ term optimally accounts for cases of 0 SE detection and can be a significant fraction of $\tilde{M}$ (∼16% at η=2 on average). Using this expression as an estimate of M lowers the variance of the denominator of eq. (1) by a factor of $e^{-\eta }$ [see Supplementary Materials].

Combining these observations, we can write the expressions for the conventional $\left({\hat{\eta }}_{\text{conv}}\right)$ and ion count-assisted (ICA, ${\hat{\eta }}_{\text{ICA}}$) estimators:

(2) $${\hat{\eta }}_{\text{conv}}=\frac{V/c_{\mu }}{\text{λ}}$$

and

(3) $${\hat{\eta }}_{\text{ICA}}=\frac{V/c_{\mu }}{{\tilde{M}}_{\text{corr}}+\text{λ}e^{-{\hat{\eta }}_{\text{ICA}}}}.$$

Note that the second of these is not a formula to evaluate but rather an equation to solve computationally. Figures S5 and S6 in the Supplementary Materials show theoretical and experimental calculations of the variance for both η estimators, demonstrating the reduction in imaging noise possible with our improved estimate of M. We also show in Figures S7 and S8 that the images produced by the conventional estimator are equivalent to those produced by the microscope software with suitable scaling.

### Images with reduced source shot noise

**Figure 2:**
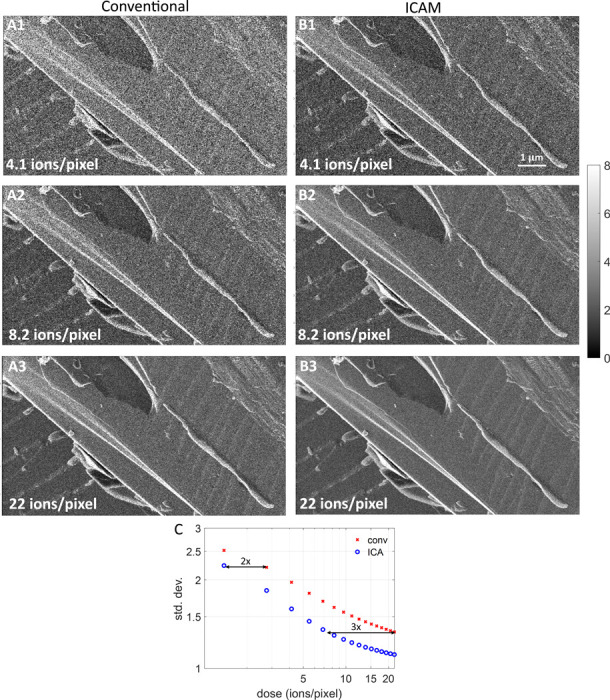
ICAM imaging of a silicon sample demonstrating source shot-noise mitigation. **(A1-A3)** Images created using the conventional SE imaging estimator at doses of 4.1, 8.2, and 22 ions/pixel. **(B1-B3)** Images created using ICAM at the same doses as the conventional images. **(C)** Standard deviation of conventional (×) and ICAM (∘) images as a function of dose. ICAM lowers the dose required to achieve a given standard deviation by a factor between 2 and 3.

Figure 2 shows comparisons between conventional and ICAM images of a scratch on a silicon chip at doses of λ=4.1, λ=8.2, and λ=22 ions per pixel. All images have 288 × 512 pixels (cropped from 512 × 512 images) and a 10 μm horizontal field-of-view. Figure S9 in the Supplementary Materials shows uncropped images of this sample at λ=22. These images are SE yield maps; instead of an arbitrary scale, gray-scale values correspond to physically meaningful values of η as indicated by the colorbar. We can see that for each dose, the ICAM images appear less noisy than the conventional images due to source shot noise mitigation. The ICAM image at a dose of 8.2 ions/pixel (Figure 2B2) appears visually similar to the conventional image at a dose of 22 ions/pixel (Figure 2A3). Figure 2C is a plot of the standard deviation measured over all the pixels as a function of the imaging dose for the conventional (×) and ICAM (∘) images. In addition to the three imaging noise components (source shot noise, target shot noise, and detector noise), this standard deviation has a contribution from variations in the features in the sample. As the dose increases, we expect all three imaging noise components to reduce, so at high doses the standard deviation should asymptotically approach the inherent feature standard deviation. This is the behavior we observe in Figure 2C. At low doses, both the conventional and ICAM standard deviations vary inversely with the dose (straight line on a log-log plot), and they show saturation at higher doses. The horizontal gaps marked in Figure 2C indicate that ICAM images have the same standard deviations as conventional images at 2- to 3-times the dose, and the doses selected for Figure 2A and B illustrate this as well.

Further evidence of the reduction in noise is provided by a calculation of Thong’s signal-to-noise ratio (SNR) for both images [32]. This metric aims to calculate SNR for a single SEM image (without knowledge of ground truth) by separating the contributions of signal and noise to the image’s autocorrelation. We calculated this SNR to be 5.6 for the ICAM image and 2.8 for the conventional image at λ=22. Figure S11 in the Supplementary Materials presents a calculation of imaging resolution using Fourier ring correlation [33, 34, 35, 36] at the 1/3 threshold [37, 38] for both images; the ICAM image shows a 21% improvement in resolution.

**Figure 3:**
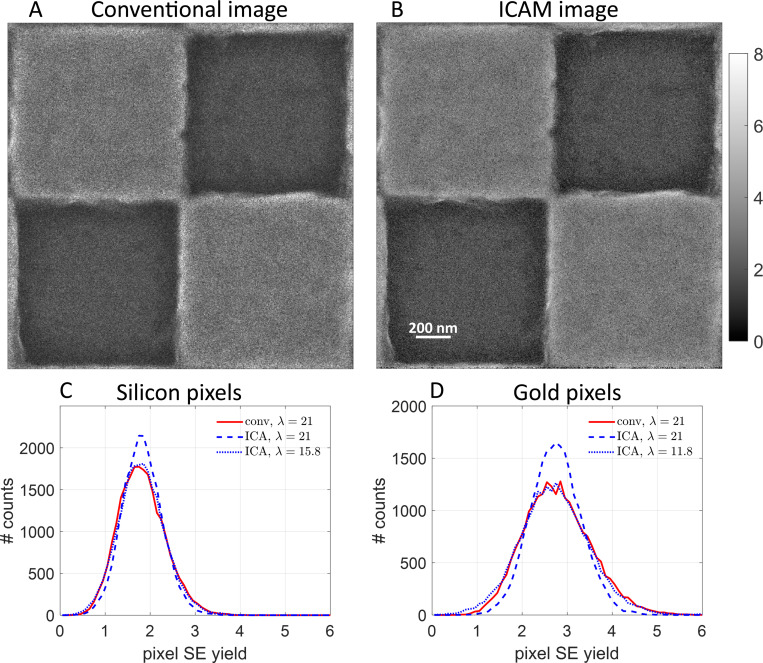
ICAM imaging of nanofabricated gold-on-silicon sample. The total field-of-view is 2 μm. **(A)** Conventional image. **(B)** ICAM image. **(C)** Histograms of pixel SE yields for darker, silicon pixels for conventional (—) and ICAM images (---) at the maximum imaging dose of λ=21. Since the histograms are computed over pixels with a common composition, their widths indicate imaging noise. ICAM imaging reduces dose requirement by a factor of 1.33, as indicated by the match between ICAM at reduced dose (···) and the conventional imaging result. **(D)** Histograms of pixel SE yields for brighter, gold pixels for conventional and ICAM images. ICAM imaging reduces dose requirement by a factor of 1.78.

Figure 3, A and B, shows the conventional and ICAM images of a patterned silicon substrate with 1 μm gold squares. The ICAM image again appears to be smoother and less noisy than the conventional image, especially in the brighter regions that correspond to gold. The lower noise in the ICAM image is also reflected in Thong’s SNR: the ICAM image has an SNR of 2.12, while the conventional image has an SNR of 1.61.

Since this sample has two types of pixels, it provides a good platform for further numerical characterization of the advantages of ICA estimation. Figure 3, C and D, are image histograms with bin width 0.1 for two subsets of pixels in this sample—the darker, silicon pixels, and the brighter, gold pixels—at the same imaging dose. The histograms plot the frequency of occurrence of different pixel SE yields. Figure 3C shows the conventional and ICAM histogram for the dark (silicon) pixels, and Figure 3D for the bright (gold) pixels. For the silicon pixels, we measured a mean SE yield of 1.82. We can see that the histogram for the ICAM image is narrower than that for the conventional image at the same dose of λ=21. The width of the ICAM histogram at a lower dose of λ=15.8 is comparable to that conventional image histogram. In other words, comparable image quality was attained using the ICA estimator at a dose that was lower than that for the conventional estimator by a factor of 1.33. For the gold pixels, we measured a mean SE yield of 2.75. The dose improvement factor in this case was 1.78.

Other performance quantifications for the images in figs. 2 and 3 also support the conclusion that ICAM improves upon conventional image formation [see Supplementary Materials].

### Theoretical predictions

**Figure 4:**
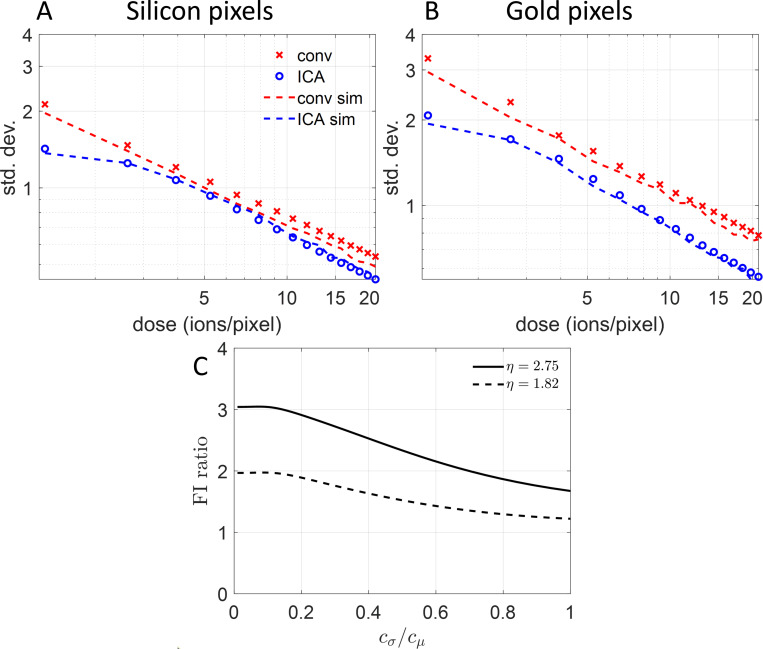
Alignment of theoretical predictions and experimental results for noise reduction due to ICA estimation. **(A)** Standard deviation of conventional and ICAM imaging as a function of imaging dose for η=1.82, corresponding to the silicon pixels in Figure 3. Note that both axes are on a logarithmic scale. The theoretical curves (--- for conventional and --- for ICAM) are close to the experimental values (× for conventional and ∘ for ICAM). **(B)** Standard deviation of conventional and ICAM imaging as a function of imaging dose for η=2.75, corresponding to gold pixels in Figure 3. **(C)** Ratio of Fisher Information for conventional and ICAM imaging for η=2.75 (—) and η=1.82 (---), as a function of $c_{\sigma }/c_{\mu }$. For the SED in our HIM, $c_{\sigma }/c_{\mu }=0.6$, and the dose reduction predicted by the FI ratio at this value closely matches our results in Figure 3.

The reduction in imaging noise in Figures 2 and 3 agrees with Monte Carlo performance predictions computed from our model of the imaging process. Figure 4, A and B, compares the theoretical standard deviation as a function of dose with the experimental values we measured for the silicon and gold pixels in Figure 3, for both conventional and ICA estimators. Unlike the plots in Figure 2C, we expect no saturation since each square in Figure 3 is almost featureless; this is exactly what we observe in Figure 4, A and B. The experimental standard deviations agree closely with the theoretical values for all doses. We also see a bigger gap between the standard deviations of conventional and ICAM images at higher SE yield, as expected from the histograms in Figure 3, C and D. As SE yield rises, an increasing fraction of incident particles produce detected SEs, making the estimate of M in eq. (3) more accurate and improving source shot noise mitigation by the ICAM estimator.

The experiments and performance predictions from simulations are consistent with theoretical analysis through Fisher information (FI). FI is a measure of the sensitivity of noisy data to the parameter to be estimated; higher FI indicates proportionately lower imaging noise. Figure 4C shows the ratio between the FI from ICAM and conventional measurement at λ=21 as a function of $c_{\sigma }/c_{\mu }$, the standard deviation of the contribution of one SE normalized by its mean, for η=2.75 (solid black curve) and η=1.82 (dashed black curve). The ratio $c_{\sigma }/c_{\mu }$ is a measure of the non-ideality of the SED—the larger this ratio, the more the SED deviates from ideal SE counting. As $c_{\sigma }/c_{\mu }$ approaches zero, the FI ratio is simply $(\eta +1)\left(1-\eta e^{-\eta }\right)$, a strictly increasing and approximately linear function of η [25]. We can see that the ratio of the FIs of ICAM and conventional measurement is greater than 1 for the entire range of $c_{\sigma }/c_{\mu }$, suggesting that ICAM images remain less noisy even for highly non-ideal detectors.

The non-ideality of the detector contributes additional noise to the image, leading to degradation in the FI ratio with increasing $c_{\sigma }/c_{\mu }$. For our system $c_{\sigma }/c_{\mu }=0.6$, and at this value we get FI ratios of about 2.1 for η=2.75 and 1.4 for η=1.82. Due to the additive property of Fisher information, we consequently expect a dose reduction by a factor of 2.1 at η=2.75 and 1.4 at η=1.82. These numbers are close to the experimental dose reductions we obtained for the sample in Figure 3.

The SE yield values we report here are not absolute, but are implicitly multiplied by the SED’s detection quantum efficiency (DQE) [39]. We measured the DQE to be about 0.88 [see Supplementary Materials]. Therefore, all SE yield values quoted here should be divided by this number for absolute SE yields. For example, the DQE-corrected mean SE yield for the sample in Figure 2 is 3.62, which is in agreement with theoretical predictions of the He-ion SE yield for silicon at 30keV [40].

Conventional estimates of SE yield require precise measurement of beam current. We observed that the beam current value measured by the picoammeter fluctuated by up to 10% for the same nominal setting of 0.11pA. As seen in eq. (2), the conventional estimate is inversely proportional to λ. Therefore, a 10% uncertainty in the value of the beam current results in a 10% uncertainty in ${\hat{\eta }}_{\text{conv}}$. In contrast, the ICAM SE yield measurement is much less sensitive to precise knowledge ofb λ because it uses the observed count of ions in addition to knowledge of λ to estimate η as seen in eq. (3). At η=2.75, a 10% variation in λ causes 0.8% variation in ${\hat{\eta }}_{\text{ICA}}$ [see Supplementary Materials]. This reduced sensitivity to precise knowledge of the beam current could be used for removal of stripe artifacts thatb result from beam current variations [41].

## Discussion and outlook

Ion count-aided microscopy differs from other techniques for image denoising and dose reduction in secondary electron imaging. Popular methods include image filtering deconvolution [17, 18, 42, 43], adaptive scanning [23], and sparse scanning and inpainting [20, 21, 22]. These methods do not attempt to model the SE generation and detection statistically, and they work as post-processing after conventional SE image generation. The ICAM method introduced here is based on statistical modelling of the SE detection process to improve initial image formation, and it can be combined with various types of post-processing.

Electron count imaging from scintillator-photomultiplier-based detectors has also been demonstrated to increase image SNR and temporal resolution in scanning transmission electron microscopy [44, 45, 46]. In this case, incident electrons that get scattered as they travel through the sample are detected. Therefore, at low beam currents, each detected pulse corresponds to one electron, similar to the case of high-energy SEM. These count-resolved methods are currently limited by pulse pile-up at higher beam currents. Since the distribution of heights of piled-up single electron pulses would be identical to that of a single multi-electron pulse, the methods presented here could be applied to model pile-up in these systems to further improve image SNR.

As shown in Figure 3, the noise-reduction factor due to the ICA estimator increases with increasing SE yield. In fig. S6B, we present theoretical calculations of the variance for η between 0 and 3.5, and we notice that noise reduction only occurs above an SE yield of about 1. This limitation would make our method less applicable to SEM at energies above ∼ 2keV. However, these estimators could still be used for low-voltage SEM, where SE yields can be high [11, 47]. Imaging with other ions, such as neon [48], would also offer higher SE yields and consequently produce lower-noise images [see Supplementary Materials]. Further, these techniques could also be used for secondary ion imaging and mass spectroscopy with helium and neon beams due to the high sputtering yield [13].

The ratio of FI of ICAM and conventional imaging, shown in Figure 4C, reduces with increasing $c_{\sigma }$, *i.e.*, increasing variance in the detector’s response to each SE single SEs. Though the large value of $c_{\sigma }/c_{\mu }$ that is typical for Everhart–Thornley detectors limits the performance of ICAM imaging, we were still able to demonstrate a significant improvement. Reduction in the variance of the SED’s response, by, for example, implementation of solid-state SE detectors [49], would improve both conventional and ICAM imaging while increasing the improvement factor of ICAM.

## Methods

### Experimental setup

We used a Zeiss Orion Plus HIM operating at 30 keV and a beam current of 0.11 pA to collect all our data. The beam current was set to the lowest stable value to reduce pulse pile-up. This beam current corresponds to a dose rate of 0.68 ions per μs. Assuming a focused probe diameter of 1 nm^2^ and a pixel dwell time of 32μs as in Figure 2 of the main paper, we get a dose of 2.2 × 10^15^ ions/cm^2^. We outcoupled the voltage signal from the SED into a Gage RazorExpress 1642 CompuScope PCIe Digitizer. In preliminary experiments to characterize the response of the SED, we observed that the SE pulses typically had a FWHM of ∼ 160 ns. We decided to use a sampling rate of 100 MS/s (corresponding to a sampling time of 10 ns) since that results in ∼ 20 samples per pulse, which we considered sufficient to ensure accurate sampling of the pulses’ peak voltages. We typically outcoupled 256 × 10^6^ samples, corresponding to a total collection time of 2.5 s. This signal was then processed using custom Matlab scripts that counted the pulses and detected their peak voltages.

### Linear Probabilistic Model for SEI

In our model, we denote the (random) number of incident ions at a given pixel by M. The ith ion generates $X_{i}$ SEs; the total number of SEs generated from the pixel is $Y=\sum_{i=1}^{M}X_{i}$. Both the number of ions M and the $X_{i}\text{s}$ are described as Poisson distributed random variables. The mean of M is known as the dose λ. The mean of $X_{i}$ is the SE yield η and is the quantity we wish to estimate. Elementary calculations give E[Y]=λη and var(Y)=λη(η+1).

Both M and the $X_{i}\text{s}$ (or, equivalently, Y) are not observed directly in the instrument. Poisson distribution since it is impossible to distinguish between an incident ion producinge We refer to the number of detected SEs as ${\tilde{X}}_{i}$, which is described by a *zero-truncated* Poisson distribution since it is impossible to distinguish between an incident ion producing zero SEs and there being no incident ion at all. Finally, ${\tilde{X}}_{i}$ is mapped to a voltage ${\tilde{U}}_{i}$. We assume that given $X_{i}=n,{\tilde{U}}_{i}$ is a Gaussian-distributed random variable described by $𝓝\left(nc_{\mu },nc_{\sigma }^{2}\right)$. Here $c_{\mu }$ and $c_{\sigma }^{2}$ are the mean and variance of the voltage produced by the SED in response to one SE, with such voltages being independent and additive. An earlier work [26] uses the less evocative $\left(c_{1},c_{2}\right)$ in place of $\left(c_{\mu },c_{\sigma }^{2}\right)$. Overall, the probability density for ${\tilde{U}}_{i}$ is given by

(4) $$f_{{\tilde{U}}_{i}}\left(u;\eta ,c_{\mu },c_{\sigma }^{2}\right)=\sum_{j=1}^{\infty }{\text{P}}_{\tilde{X}}(j;\eta )f_{Z}\left(u;j,c_{\mu },c_{\sigma }^{2}\right)\;\;=\sum_{j=1}^{\infty }\frac{e^{-\eta }}{1-e^{-\eta }}\frac{\eta^{j}}{j!}f_{Z}\left(u;j,c_{\mu },c_{\sigma }^{2}\right),$$

where $f_{Z}\left(u;j,c_{\mu },c_{\sigma }^{2}\right)$ is the PDF of a $𝓝\left(jc_{\mu },jc_{\sigma }^{2}\right)$ random variable. We assume that λ, $c_{\mu }$, and $c_{\sigma }$ are known.

The assumption of linearity in our detection model will hold as long as pulse pile-up is small. At small levels of pile-up, the correction term to the raw pulse counts works well. At beam currents significantly higher than those used in this study, we expect that more careful consideration will need to be given to the statistics of overlapping pulses, which would lead to improved estimators.

### Measurement of SED response parameter $c_{\mu }$

To characterize the detector’s response to SEs and measure $c_{\mu }$, we imaged a featureless silicon chip to ensure that SE emission was uniform over the scan region. Further, we defocused the beam by 5mm to reduce beam-induced damage and mitigate variations in SE yield η due to local contamination or topography. Since $c_{\mu }$ and $c_{\sigma }$ are properties of the detector, we expect them to remain unchanged with variations in the sample; i.e., our measurements of $c_{\mu }$ and $c_{\sigma }$ should be constant for samples with variable η. Therefore, we found it useful to be able to continuously vary η to validate our measurements. This variation of η would be difficult to accomplish with physical samples; instead, we varied the collector bias on the Everhart–Thornley SED to change the effective SE yield at the detector. Everhart–Thornley detectors typically have a positively-biased metal cage at the front to attract SEs. The default bias on the cage was 500 V; we varied this collector bias between 0V and 500 V to get different effective values of η.

In our model, $c_{\mu }$ is the mean voltage produced by the detector per SE. Therefore, the most direct method for measuring $c_{\mu }$ would be to have exactly one SE incident on the detector many times and compute the mean voltage produced by the SED. Unfortunately, such deterministic irradiation of the SED is not possible, since the production of SEs is a Poisson process. One could imagine placing the SED under an electron beam produced by an electron gun, as was done in [29]. However, such an experiment requires significant modification of the microscope, which was not possible on our tool.

Instead, we measured $c_{\mu }$ by imaging a sample with low SE yield η. At sufficiently low SE yield, the probability that the incident particle will excite more than one SE is small. Therefore, we can assume that most detected pulses are excited by one SE. Under these conditions, if our model is accurate, we would expect the *pulse height distribution (PHD)*, i.e., the histogram of the peak voltages of the SE pulses, to be approximately Gaussian with a peak at $c_{\mu }$. Using this technique, we extracted $c_{\mu }=0.163\;\text{V}$ from a histogram measured at $\hat{\eta }=0.58$. In the Supplementary Materials, we describe the use of the probabilistic model to fit experimental PHDs at various values of $\hat{\eta }$.

### Correcting $\tilde{M}$ for pulse pile-up

As discussed in the paper, ions arriving consecutively in a short time frame can generate overlapping pulses, a phenomenon known as pulse pile-up. Consequently, the number of f local maxima in a voltage signal can be an underestimation of $\tilde{M}$. One way to correct for missed detections due to pile-up is by introducing a multiplicative factor:

(5) $${\tilde{M}}_{\text{corr}}=\frac{\tilde{M}}{\gamma_{\tau }(\text{Λ},\eta )},$$

where

(6) $$\gamma_{\tau }(\text{Λ},\eta )=\exp\left(-\text{Λ}\left(1-e^{-\eta }\right)\tau \right)$$

is the probability that two pulses arrive within time τ of each other, and $\text{Λ}=\text{λ}/t_{d}$ is the dose per unit time. We chose τ=0.13μs; more details on the choice of τ can be found in the Supplementary Materials.

## Supplementary Material

Supplement 1

## Data Availability

Raw data used to produce all images and graphs herein are available at https://doi.org/10.5281/zenodo.10535916.
