# Supplementary material for: Shot noise-mitigated secondary electron imaging with ion count-aided microscopy
Source: ArXiv. 2024 Jul 8:arXiv:2311.07003v2. Preprint. [Version 2] (PMC11261977)
Supplement: Supplement 1 [file NIHPP2311.07003v2-supplement-1.pdf]

arXiv:2311.07003v2 [physics.med-ph] 8 Jul 2024

Supplementary Materials for

Shot Noise-Mitigated Secondary Electron Imaging with Ion Count-Aided Microscopy

Akshay Agarwal *et al.*

Corresponding authors: Akshay Agarwal, akshayag@bu.edu; Vivek K Goyal, v.goyal@ieee.org

This PDF file includes:

Supplementary text
Figures S1 to S13

Supplementary Text

Contents

- Section S1: Distribution of pulse interarrival times.
- Section S2: Fitting pulse height distributions using η estimates.
- Section S3: Design and analysis of estimators for ion count M and SE yield η.
- Section S4: Comparison between our conventional images and images from instrument software.
- Section S7: Further quantitative analysis through signal-to-noise ratio and resolution.
- Section S8: Computation of detector quantum efficiency.
- Section S9: Analysis of sensitivity of estimators to beam current fluctuation.
- Section S10: Analysis of relative error dependence on scaling of SE yield.

S1 Distribution of pulse interarrival times

Figure S1A shows a 50 µs example of the outcoupled voltage signal. As preliminary evidence for our probabilistic model, Figure S1B shows a histogram of the interarrival times between successive pulses for the same dataset as Figure S1A. This data was obtained from the silicon sample by setting the SED collector bias to 0 V. If $\widetilde{M}$ follows a Poisson distribution as discussed in Section S3.5, the interarrival times should be exponentially distributed with parameter $\lambda(1-e^{-\eta})$. As indicated by the linearity of the interarrival time distribution on the semilog plot in Figure S1B, the interarrival times indeed follow an exponential distribution. The parameter of the distribution was obtained from a linear fit (solid orange line) to be 0.275 per µs. With $\lambda = 0.625$, we use eq. (S13) to get $\widehat{\eta}_{\widetilde{M}} = 0.58$. Such estimates provide a useful benchmark for the characterization of other ICA estimators, as detailed in Section S3.6.

S2 Fitting pulse-height distributions using η estimates

We fitted the distribution of pulse heights at different values of η to the probabilistic model described in Methods. This fitting yields the parameter c_σ, the variance of the SED's response to one SE. While c_σ does not enter into estimate computations, it is critical in performance predictions.

Figures S2 and S3 show the experimental PHD (○) and the voltage probability distribution from our model (—) for various values of η. Recall that the value of η was varied by changing the SED collector bias (from 0 V for the lowest

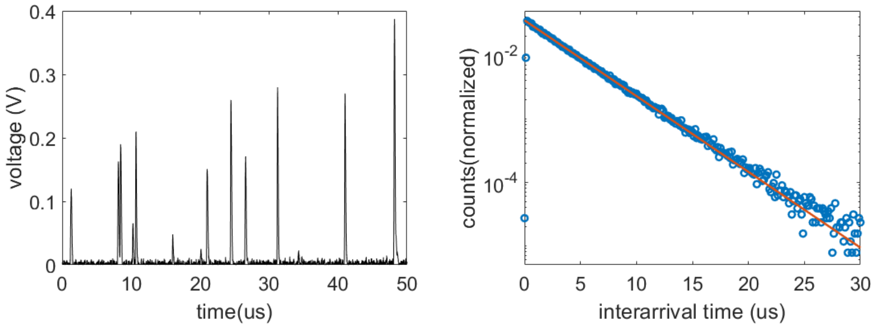

(A) SED signal (B) Inter-arrival time distribution

Figure S1: Initial characterization of SED signal. The interarrival time distribution follows an exponential distribution and allows estimation of η.

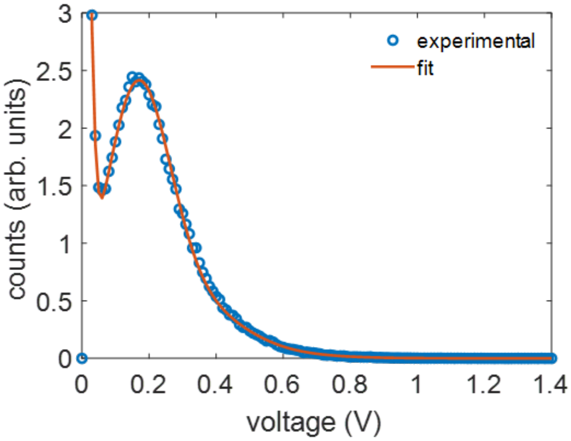

(A) Linear, $\widehat{\eta}_{\text{ICA}} = 0.58$

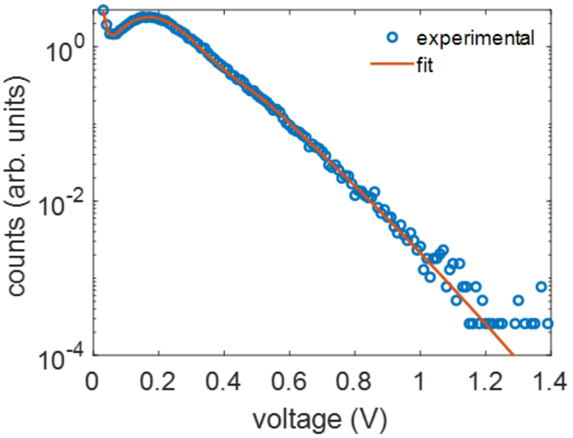

(B) Log, $\widehat{\eta}_{\text{ICA}} = 0.58$

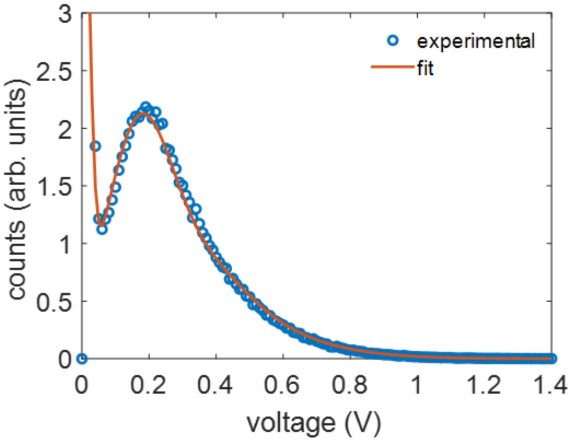

(C) Linear, $\widehat{\eta}_{\text{ICA}} = 1.1$

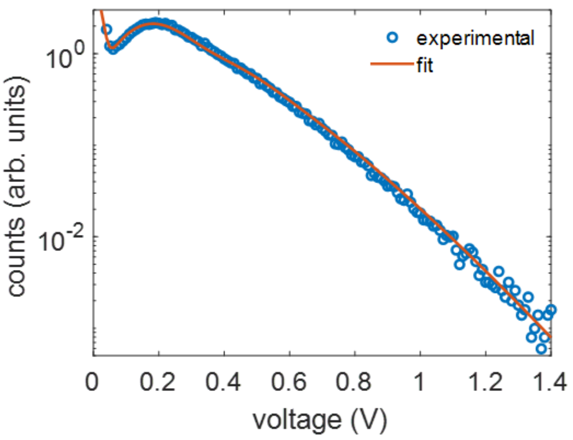

(D) Log, $\widehat{\eta}_{\text{ICA}} = 1.1$

Figure S2: Pulse height distribution (blue circles) and model fit (solid orange line) for various values of η on linear (left) and log (right) scale. Caption indicates the ICA and fit η values. In all cases, the fits used $c_\mu = 0.163\ V$ ad $c_\sigma = 0.097\ V$.

η to 500 V for the highest); the physical sample is silicon in all cases. For each value of η, the left panel shows the PHD and model fit on a linear scale, and the right panel shows the same fits on a log scale. The figure captions also indicate the ICA estimate of η used for these fits. We note that in obtaining these fits, we added a zero-mean Gaussian component to fit the low-voltage noise in the PHD. For all fits, this component had a relative amplitude between 0.76 and 0.78 compared to the signal, and a constant standard deviation of 0.02. We see that for all values of η, the PHD is very well described by the model with $c_\mu = 0.163\,\text{V}$ and $c_\sigma = 0.097\,\text{V}$. For low values of η (Figure S2), the entire PHD is fit by the model. At higher values of η (Figure S3), a high-voltage feature emerges in the PHD around 1.6 V, which causes deviations between the PHD and the model fit. This feature can be seen most clearly in the log plots. We expect that this feature occurs because of pulse height saturation. The maximum voltage the SED produced in all of our experiments was 1.8 V, which corresponds to about 11 SEs. As η increases, the probability of producing more than 11 SEs increases, and these pulses get saturated at or near 1.8 V causing the feature seen in the PHD. Inclusion of such saturation in our model would improve the fits at higher values of η.

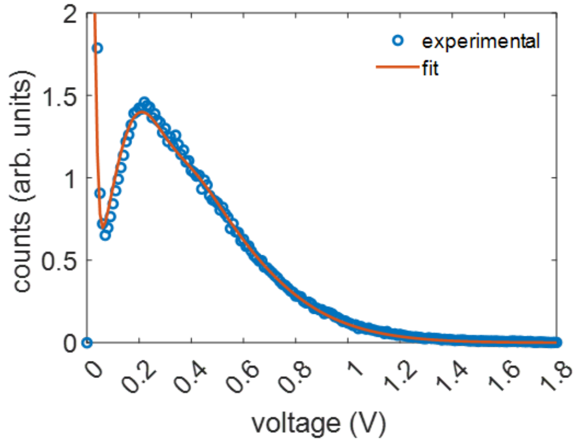

(A) Linear, $\widehat{\eta}_{\mathrm{ICA}} = 2.1$

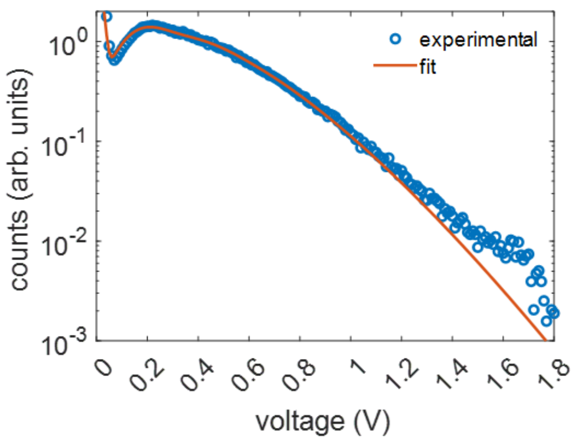

(B) Log, $\widehat{\eta}_{\mathrm{ICA}} = 2.1$

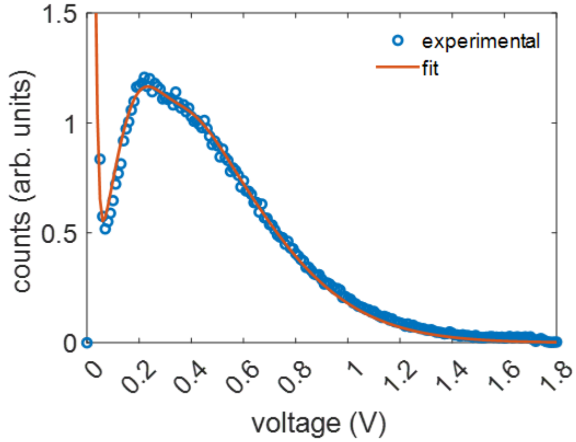

(C) Linear, $\widehat{\eta}_{\mathrm{ICA}} = 2.57$

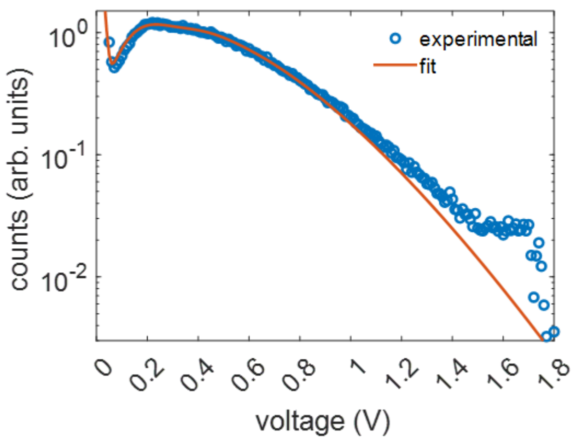

(D) Log, $\widehat{\eta}_{\mathrm{ICA}} = 2.57$

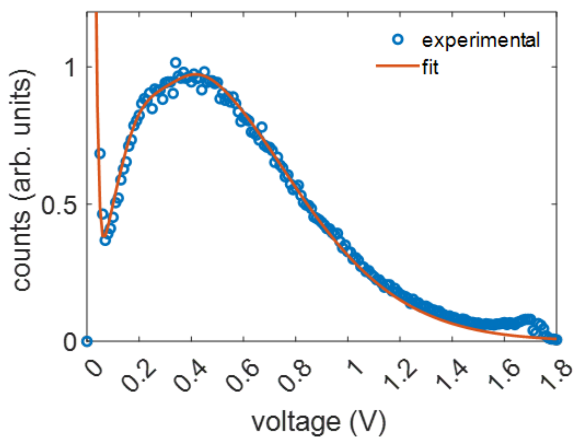

(E) Linear, $\widehat{\eta}_{\mathrm{ICA}} = 3.25$

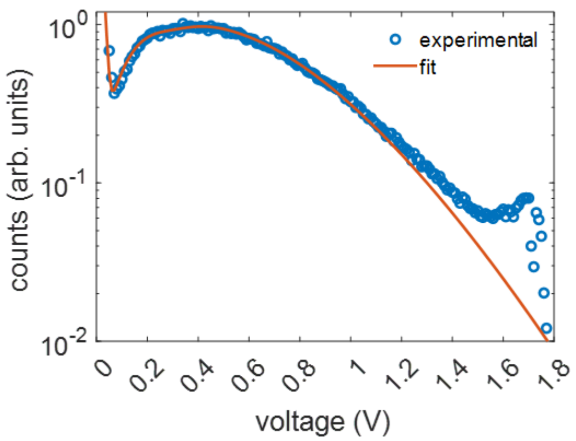

(F) Log, $\widehat{\eta}_{\mathrm{ICA}} = 3.25$

Figure S3: Same as Figure S2 for higher values of η. Deviation between model and experimental PHD can be seen at higher voltages due to pulse saturation.

S3 Estimators for Ion Count M and SE Yield η

As discussed in the paper, an intuitive form for SE yield estimators is

$$\widehat{\eta} = \frac{\text{number of SEs detected}}{\text{number of incident particles}}. \tag{S1}$$

In this section, we introduce and analyze several estimators of this form. They differ in how the ion count M is estimated for use in the denominator of eq. (S1). Since some estimators for M have dependence on η, which is unknown, the substitution can yield an equation to solve for $\widehat{\eta}$.

S3.1 Oracle-aided estimator

If Y and M were provided by an oracle, with $M > 0$, η could be estimated as

$$\widehat{\eta}_{\text{oracle}} = \frac{Y}{M}. \tag{S2}$$

Conditioned on M being greater than 0, this is an unbiased and efficient estimate of η. Though not implementable from measurements we have available, it will serve as a useful theoretical bound on the performance of other, more practical, estimators.

S3.2 Conventional estimator

For estimating Y, suppose the sum of the peak voltages from all the pulses generated by the ions incident on the pixel within the dwell time is $V = \sum \widetilde{U}_i$. Then, $V \sim \mathcal{N}(c_\mu Y, c_\sigma^2 Y)$. Thus, V/c_μ is the maximum likelihood (ML) estimator for Y, and it has conditional variance $c_\sigma^2 y / c_\mu^2$ given $Y = y$. As discussed in the paper, λ is an unbiased estimate of M. Therefore, the variance (and mean-squared error (MSE)) of this estimate is given by

$$\mathbb{E}\left[\, (M - \lambda)^2 \,\right] = \lambda. \tag{S3}$$

Substituting these estimates into eq. (S1), we arrive at the *conventional* estimator:

$$\widehat{\eta}_{\text{conv}} = \frac{V/c_\mu}{\lambda}. \tag{S4}$$

It is unbiased and has MSE

$$\mathbb{E}\left[\, (\eta - \widehat{\eta}_{\text{conv}})^2 \,\right] = \frac{\eta(\eta + 1 + c_\sigma^2/c_\mu^2)}{\lambda}. \tag{S5}$$

This MSE expression shows the contribution from SED noise ($c_\sigma > 0$). Having quadratic (rather than linear) dependence on η is a manifestation of source shot noise [1].

We note that the form of this estimator differs from the way SE imaging is conventionally performed. Typically, SEI does not aim to directly estimate η; instead, the aim is to create an image that qualitatively represents variations in η over the sample. Therefore, eq. (S1) is not directly involved in conventional imaging. Instead, the voltage waveform is sampled at a constant rate. Each of the detected pulses has a FWHM of $\sim 160\,\text{ns}$, and the sampling time needs to be lower than this width to ensure that each pulse is accurately sampled. In practice, a sampling time of $100\,\text{ns}$ is typically used. This sampled voltage is quantized to (usually) 8 bits by an ADC. Other unspecified operations may also be applied to the SED voltage signal before quantization. The final image is a map of this 'pixel brightness'.

We found that our conventional estimator and 'true' conventional imaging had almost identical performance in terms of imaging noise, after the true conventional image was appropriately scaled. In Section S4, we show images created by both methods; their appearances and histograms are close to identical. Therefore, it is appropriate to use the conventional estimator to represent the performance of existing SEI, as done in the main paper.

S3.3 Quotient-mode estimator

In the previous section, we described how V/c_μ is the ML estimator for Y. We also found the variance for using λ as an estimate of M to be equal to λ. To improve the estimation of M, we notice that in addition to the peak voltage of each pulse, we also know the total number of detected pulses, $\widetilde{M}$. Since each pulse corresponds to events from one incident ion, we can use $\widetilde{M}$ as an estimate of M. We note that this estimator will be biased, since it does not account for the cases where an incident ion produces 0 SEs, and therefore it underestimates M. The MSE for this estimator is given by

$$\mathbb{E}\Big[(M - \widetilde{M})^2\Big] = \lambda e^{-\eta}(1 + \lambda e^{-\eta}). \tag{S6}$$

For large η, this expression becomes very small, indicating that the MSE from this estimate will be lower than that in eq. (S3). However, for small η, this MSE is approximately $\lambda(1 + \lambda)$, which is larger than that in eq. (S3).

Using $\widetilde{M}$ as an estimator for M, we get the *quotient-mode* (QM) estimator:

$$\widehat{\eta}_{\text{QM}} = \frac{V/c_\mu}{\widetilde{M}}. \tag{S7}$$

The accuracy of $\widetilde{M}$ as an estimate of M given in eq. (S6) is reflected in the MSE of $\widehat{\eta}_{\text{QM}}$. At low η, many incident ions produce 0 SEs, and therefore $\widetilde{M}$ significantly underestimates M and results in high MSE. At high η, almost all incident ions produce at least one detected SE, and therefore $\widetilde{M}$ is an accurate estimate of M and results in low MSE. An abandoned patent application [2] contains a description similar to the quotient-mode estimator, though this was apparently not implemented in any product. This may be due to its poor performance at low η.

S3.4 Maximum likelihood-inspired estimator

Inspired by the QM estimator, we could try to improve upon $\widetilde{M}$ as an estimate of M. Such an estimator should depend on $\widetilde{M}$, but must also somehow include a correction for the cases in which 0 SEs are detected. Since the mean number of incident ions is λ, and the number of SEs emitted per ion has a Poisson distribution with mean η, the expected number of ions that result in 0 SEs is $\lambda e^{-\eta}$. Therefore, $\widetilde{M} + \lambda e^{-\eta}$ should be an improved, unbiased estimator for M. Indeed, through more rigorous arguments detailed in [3], this quantity is the minimum MSE estimator for M. The MSE for this estimator can be calculated as

$$\mathbb{E}\Big[(M - (\widetilde{M} + \lambda e^{-\eta}))^2\Big] = \lambda e^{-\eta}, \tag{S8}$$

which is uniformly lower than the expression in eq. (S3) by a factor of $e^{-\eta}$. Using this estimate of M in eq. (S1), we get

$$\widehat{\eta}_{\text{MLI}} = \frac{V/c_\mu}{\widetilde{M} + \lambda e^{-\widehat{\eta}_{\text{MLI}}}}. \tag{S9}$$

This equation can be solved with a suitable root-finding algorithm. We refer to this estimator as *ML-inspired* (MLI), rather than ML, because a true ML estimator would require maximization of the likelihood for the joint observation $(V, \widetilde{M})$ under the probabilistic model described in Methods. Such an estimator cannot be expressed using a simple analytical expression. Because we are using efficient estimators for both Y and M, we expect the performance of $\widehat{\eta}_{\text{MLI}}$ be close to a true ML estimator, and we confirmed through Monte Carlo simulations that this is indeed the case. This estimator is also more computationally tractable than a true ML estimator for large datasets.

Correcting the MLI estimator for pulse pile-up: As described in Methods, we accounted for pulse pile-up by introducing $\widetilde{M}_{\text{corr}}$:

$$\widetilde{M}_{\text{corr}} = \frac{\widetilde{M}}{\gamma_\tau(\Lambda, \eta)}, \tag{S10}$$

where

$$\gamma_\tau(\Lambda, \eta) = \exp(-\Lambda(1 - e^{-\eta})\tau) \tag{S11}$$

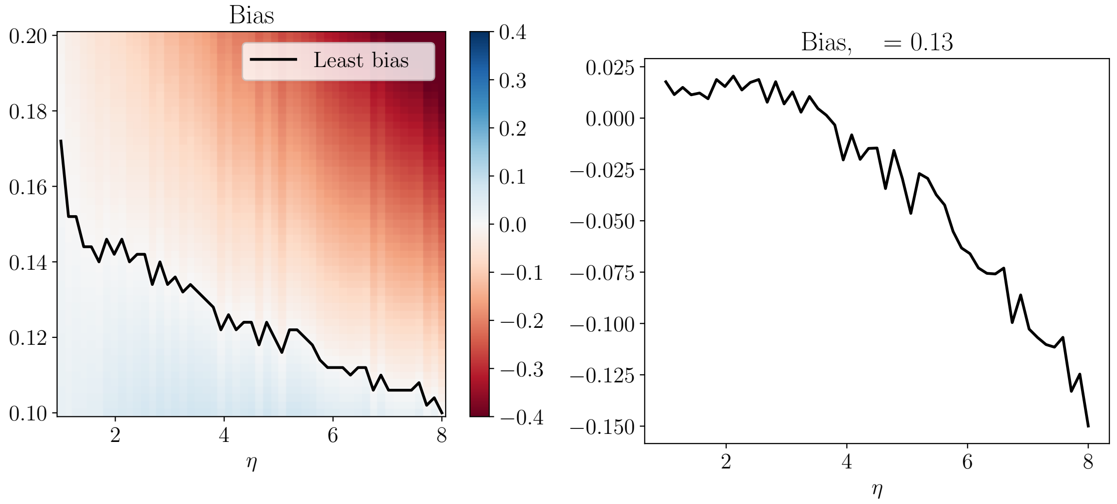

(A) Bias at different η and τ

(B) Bias at $\tau = 0.13$

Figure S4: Bias of the count-corrected ML-inspired estimator $\widehat{\eta}_{\text{CCMLI}}$, defined in eq. (S12), obtained through Monte Carlo simulation with 10 000 trials. The rate Λ is 0.625 ion/µs. The line in (A) is τ at which the absolute bias is minimized at each η.

is the probability that two pulses arrive within time τ of each other, and $\Lambda = \lambda/t_d$ is the dose per unit time [3]. Substituting $\widetilde{M}$ by $\widetilde{M}_{\text{corr}}$ in eq. (S9) yields the *count-corrected ML-inspired* estimator:

$$\widehat{\eta}_{\text{CCMLI}} = \frac{V/c_\mu}{\widetilde{M}/\gamma_\tau(\Lambda, \widehat{\eta}_{\text{CCMLI}}) + \lambda e^{-\widehat{\eta}_{\text{CCMLI}}}}. \tag{S12}$$

One remaining challenge is choosing an appropriate τ. In addition to the inter-arrival time, both widths and heights of the pulses determine whether a given set of pulses overlap and merge into one large pulse. The pulses have randomly varying widths and heights, the latter of which depends on the number of SEs generated, which further depends on unknown η. Nevertheless, as shown in Figure S4, choosing τ around 0.12 µs to 0.14 µs yields an estimator $\widehat{\eta}_{\text{CCMLI}}$ with a small bias for $\eta \in [1, 8]$, a suitable range of interest for HIM. These plots were computed using a Monte Carlo simulation whose details are described in Section S3.6. For all the results in the paper, we chose $\tau = 0.13$ µs. Figure S4B shows the bias in the CCMLI estimator for this choice of τ; in absolute value, it remains below 0.02 for the range of η imaged in the paper.

The count-corrected MLI estimator was used to compute all the ICAM images in the paper. In the paper, the estimator is referred to simply as the ion count-aided estimator $\widehat{\eta}_{\text{ICA}}$ because we do not wish to compare different ion count-aided estimators there (one could argue that $\widehat{\eta}_{\text{QM}}$ and $\widehat{\eta}_{\text{MLI}}$ without count correction are also ion count-aided estimators because they use knowledge of $\widetilde{M}$). In the subsequent sections of this document, we return to the use of $\widehat{\eta}_{\text{ICA}}$ to refer to this estimate, which incorporates our modeling insights without introducing high computational complexity through full use of the probabilistic model or the possibility of an η-dependent τ value.

S3.5 Pulse-count estimator

All the practically implementable η estimators described so far require knowledge of c_μ, the mean voltage produced by 1 detected SE. However, it would be good to validate the η estimates from these estimators against an independent measurement of η that does not rely on knowledge of c_μ. We could potentially measure η indirectly from the sample current from the silicon sample, but such a measurement cannot be expected to agree with η measured at the SED due to non-ideal detection quantum efficiency (DQE) [4, 5].

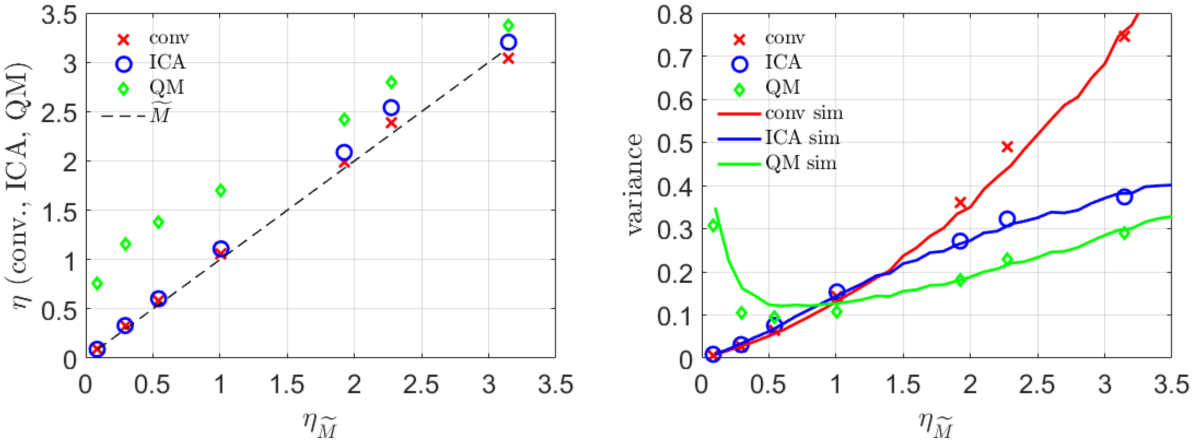

(A) Mean vs. η (B) Variance vs. η

Figure S5: Performance of ICA, QM, and conventional η estimators for different values of η. The mean values of the ICA and the conventional estimators agree with the baseline $\widehat{\eta}_{\widetilde{M}}$, while the variance of the ICA estimator is lower than that of the conventional estimator for $\eta > 1$. The QM estimator's variance is lower than the ICA, but it has a large bias.

Instead, we will use just the count of SE pulses $\widetilde{M}$ to estimate η. Since M is modeled as $\text{Poisson}(\lambda)$ and the count is thinned by ion incidences that produce no detected SEs, $\widetilde{M} \sim \text{Poisson}(\lambda(1 - e^{-\eta}))$. If we observe $\widetilde{M}$ pulses, we can estimate η as

$$\widehat{\eta}_{\widetilde{M}} = -\log\left(1 - \frac{\widetilde{M}}{\lambda}\right). \tag{S13}$$

The properties of this estimator are discussed in [3]. Briefly, we expect this estimator to be accurate at low η, where it is very likely to have $\widetilde{M} < \lambda$. At high η, almost all the incident ions will produce at least one SE. Therefore, $\widetilde{M}$ will be pretty close to λ and might even exceed it if $M > \lambda$ in some experiments. The estimate in eq. (S13) is likely to be highly inaccurate in this case. However, at low η, the estimator will be accurate, and we will use it to benchmark the results of other estimators in Section S3.6.

S3.6 Validation and comparison of estimators on bulk samples

We used the measured c_μ to implement the conventional, QM, and ICA SE yield estimators. As discussed earlier, we used $\widehat{\eta}_{\widetilde{M}}$ as a benchmark for the other η estimators at low η, where $\widehat{\eta}_{\widetilde{M}}$ is reliable. This benchmarking allowed us to be more confident in the η estimators at high η, where $\widehat{\eta}_{\widetilde{M}}$ is not reliable. As discussed earlier, we collected 2.5 s of data from featureless silicon samples, and we varied the effective η by changing the SED collector bias. For the results described below, we used a pixel dwell time of 25 µs and a beam current of 0.11 pA, resulting in $\lambda = 17.2$ ions/pixel. For counting pulses and measuring their peak voltages, we used a threshold voltage of 0.07 V to filter out noise peaks.

Figure S5 shows the performance of the QM, ICA, and conventional estimators for η values between 0 and 3.2. In Figure S5A, we compare the benchmark measurement of η, *i.e.*, $\widehat{\eta}_{\widetilde{M}}$ (- - -), with the conventional (○), ICA (×), and QM (◇) estimates. We see that the conventional and ICA estimates are close to the benchmark values for the whole range of η. It should be noted that $\widehat{\eta}_{\widetilde{M}}$ becomes increasingly unreliable as η increases as discussed earlier, and therefore the differences between $\widehat{\eta}_{\widetilde{M}}$ and the other estimators at high η might be due to inaccuracies in $\widehat{\eta}_{\widetilde{M}}$. The QM estimator shows a significant bias at small η, as we would expect, and converges somewhat to the conventional and ICA estimators at higher η. The agreement between the benchmark and the conventional and ICA estimators is strong validation for our abstract model of SE generation.

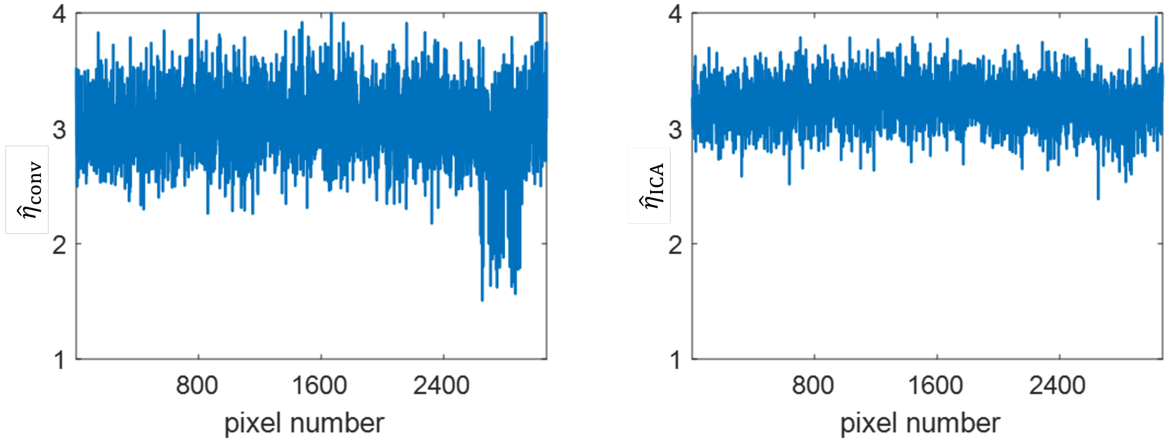

(A) $\widehat{\eta}_{\text{conv}}$ (B) $\widehat{\eta}_{\text{ICA}}$

Figure S6: One-dimensional imaging of a uniform silicon sample. The conventional estimate has higher variance (0.091) than the ICA estimate (0.036), resulting in a noisier reconstruction of η.

Figure S5B shows the variance of the conventional, ICA, and QM estimators vs. η. The plot also shows theoretical curves for the variances of each of these estimators. These curves were generated using Monte Carlo simulations of the signal produced by the SED using our model described at $\lambda = 17.2$ ions. The voltage pulse from each burst of SEs was modelled as a Gaussian in the time domain, with FWHM of $160\,\text{ns}$. Pulse heights and counts were extracted from this synthetic detector voltage provided as inputs to the η estimators described in the previous sections. The variances of the estimators are in line with theoretical expectations; the ICA estimator has lower variance than conventional for η greater than ~ 1. As an example, the variance for the ICA estimator is lower than that of the conventional by a factor of 2 at $\eta = 3$. Since the variance is inversely proportional to imaging dose, we would expect a factor of 2 reduction in the imaging dose required for a given noise level compared to conventional imaging. This factor of dose reduction is exactly what we had observed in the results presented in Figure 3 of the main paper. The QM estimator has even lower variance than the ICA estimator at high η, but, as already discussed, has a relatively high bias, too. Overall, the lower variance of the ICA estimator is experimental evidence of its success in lowering noise in imaging.

Figure S6 presents a different perspective on the reduction in variance from the ICA estimator. Figure S6A is the conventional estimate of η in a one-dimensional scan over the uniform silicon sample (at an SED voltage bias of 500 V). The mean value of η here is 3.15. Since the sample is uniform, all variations in η are due to randomness in M, Y, and V; the variance of the estimate is 0.091. Figure S6B is the ion count-aided estimate of η using the same dataset. We can see that the noise in this estimate is lower than the conventional estimate; the variance of the ICA estimate is 0.036. Lower imaging noise results in increased resolution at the same dose.

S4 Similarity of Conventional Estimator and Software Images

As discussed in the main paper, we used the conventional estimator to represent typical SEI. Figures S7 and S8 show two examples of images produced by our conventional estimator alongside images produced by the SEI software on the microscope. For the purposes of this comparison, we scaled the conventional image to have the same mean as the software image, since the two images are originally on different scales. Figure S7 compares the (A) software and (B) conventional images of the same sample that was used in Figure 2 in the paper. Note that due to the rescaling of the conventional image, the grayscale does not reflect SE yields anymore. We can see that the two images appear to be very similar. The visual similarity is confirmed by the image histograms of the two images in Figure S7C. Figure S8 compares the software and conventional images of a sample of agglomerated silver nanoparticles. Again, the two images and their histograms are nearly identical.

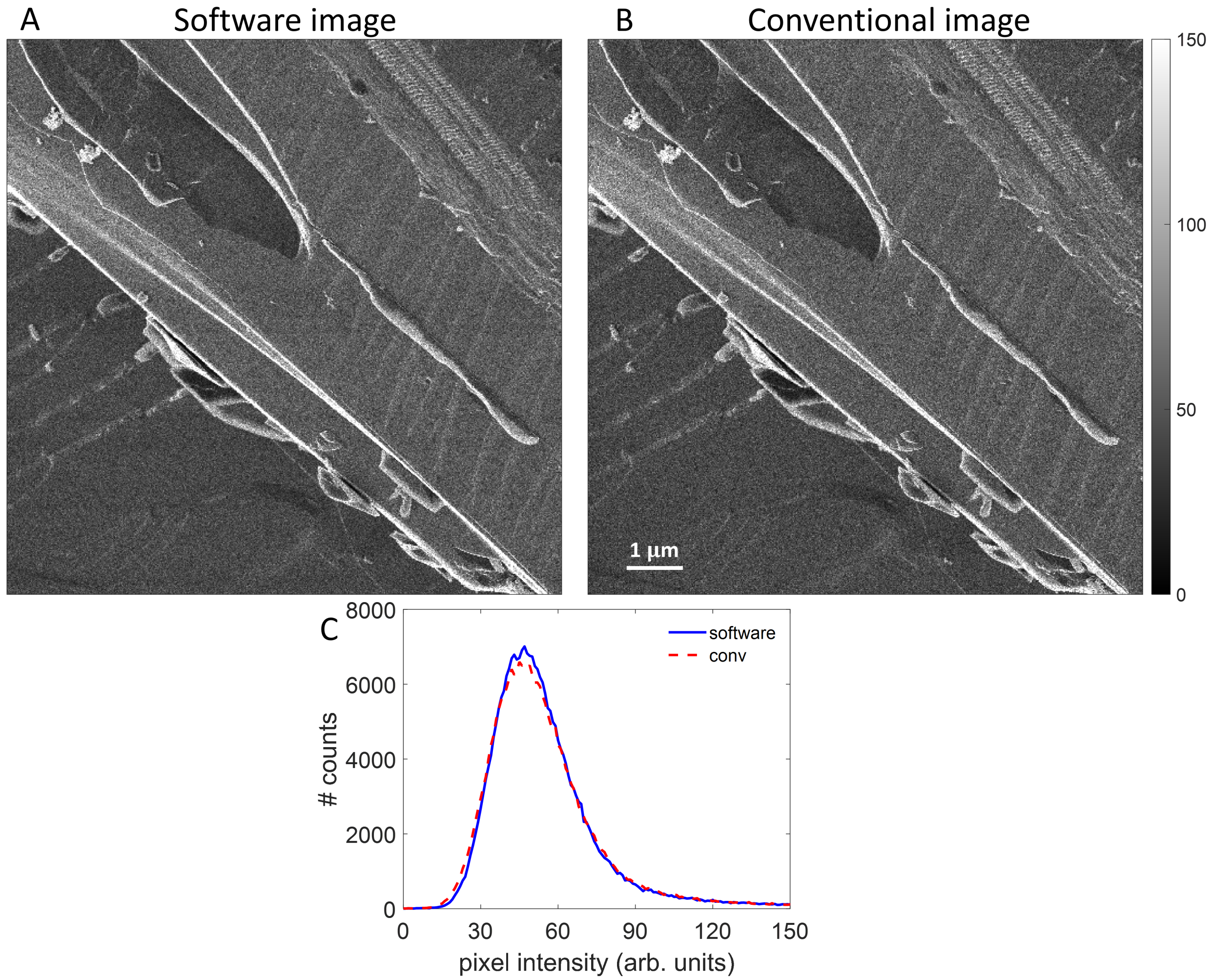

Figure S7: Conventional and software images of a scratch on silicon. (A) Image generated by microscope imaging software. (B) Image generated using conventional estimator. The images are visually similar. (C) Pixel intensity histograms of the two images. The two histograms are nearly identical.

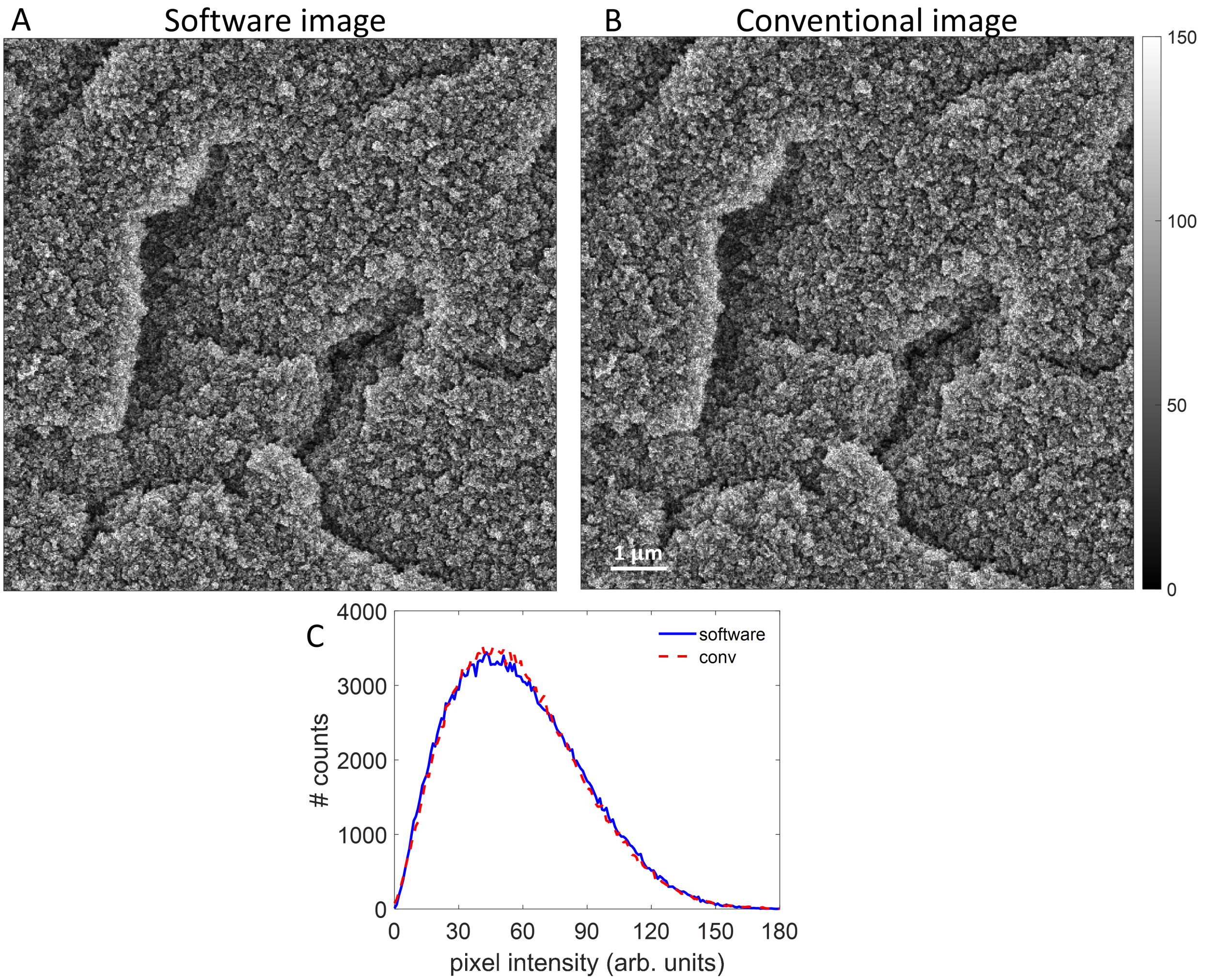

Figure S8: Conventional and software images of agglomerated silver nanoparticles. (A) Image generated by microscope imaging software. (B) Image generated using conventional estimator. The images are visually similar. (C) Pixel intensity histograms of the two images. The two histograms are nearly identical.

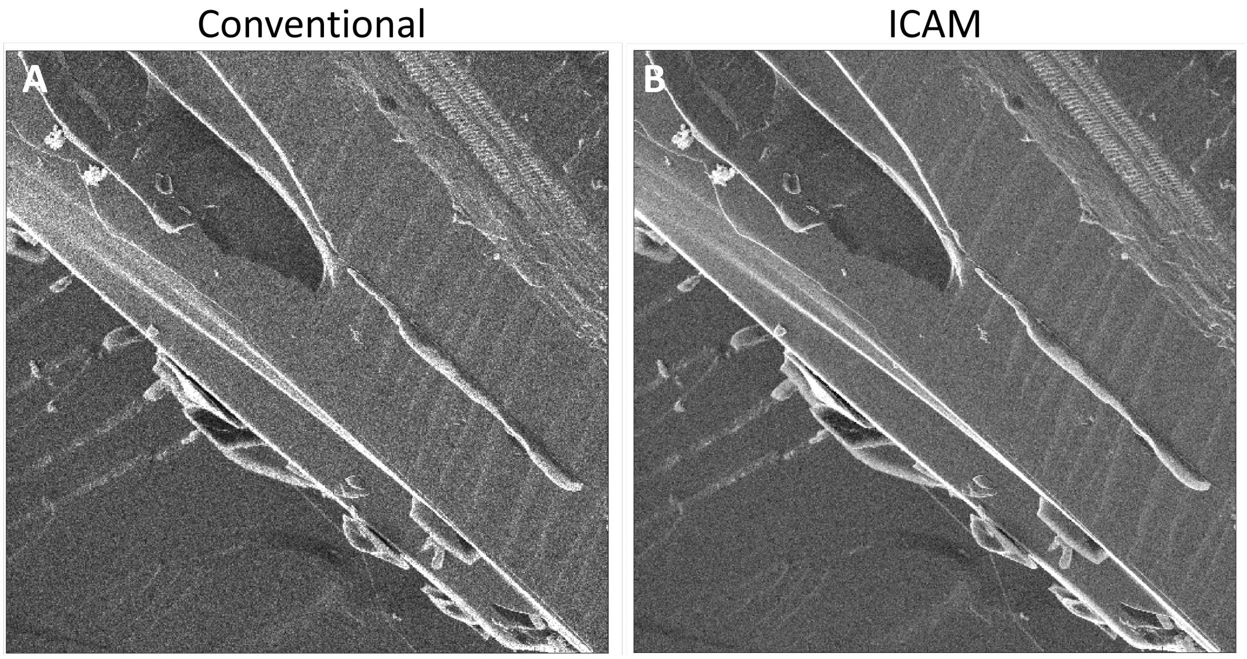

Figure S9: Uncropped images of the sample imaged in Figure 2 at dose $\lambda = 22$ ions. (A) Conventional image (B) ICAM image.

S5 Uncropped Images for Figure 2

Figure S9 shows uncropped conventional and ICAM images of the same silicon sample used to create Figure 2 in the paper.

S6 Performance of conventional and ICAM imaging at high magnification

Figure S10A and B shows an exmaple of conventional and ICAM imaging at a field of view of 1 μm, respectively. Figure S10C is a plot of the image standard deviation as a function of dose for the two imaging modes. We can see that ICAM achieves reduction in dose (between a factor of 2 and 3) for a given standard deviation. This gain is similar to that reported in the paper at a field of view of 10 μm. This result, along with the agreement between theoretical and experimental predictions in Figure 4 of the paper, show that the gains offered by ICAM are independent of image magnification.

S7 Performance Metrics for Conventional and ICAM Images

Silicon scratch sample: In the paper we calculated Thong's SNR metric for the conventional and ICAM images of the silicon scratch sample. In addition to this metric, we can also calculate SNR using Saxton's method [6]. Saxton proved that the SNR could be computed from the normalized cross-correlation of two independently acquired, zero-mean images of a ground truth sample. To implement this SNR metric, we divided our dataset into two halves, and we created conventional and ICAM images from each half-dataset independently. Next, we computed the normalized cross-correlation at zero offset between these half-dataset images after subtracting their means, and used the result to compute the SNR. We repeated this calculation for 1000 different half-dataset divisions of the full dataset. We obtained an average SNR of 1.17 for the conventional image, and 2.11 for the ICAM image, again indicating an SNR improvement by a factor of 2.

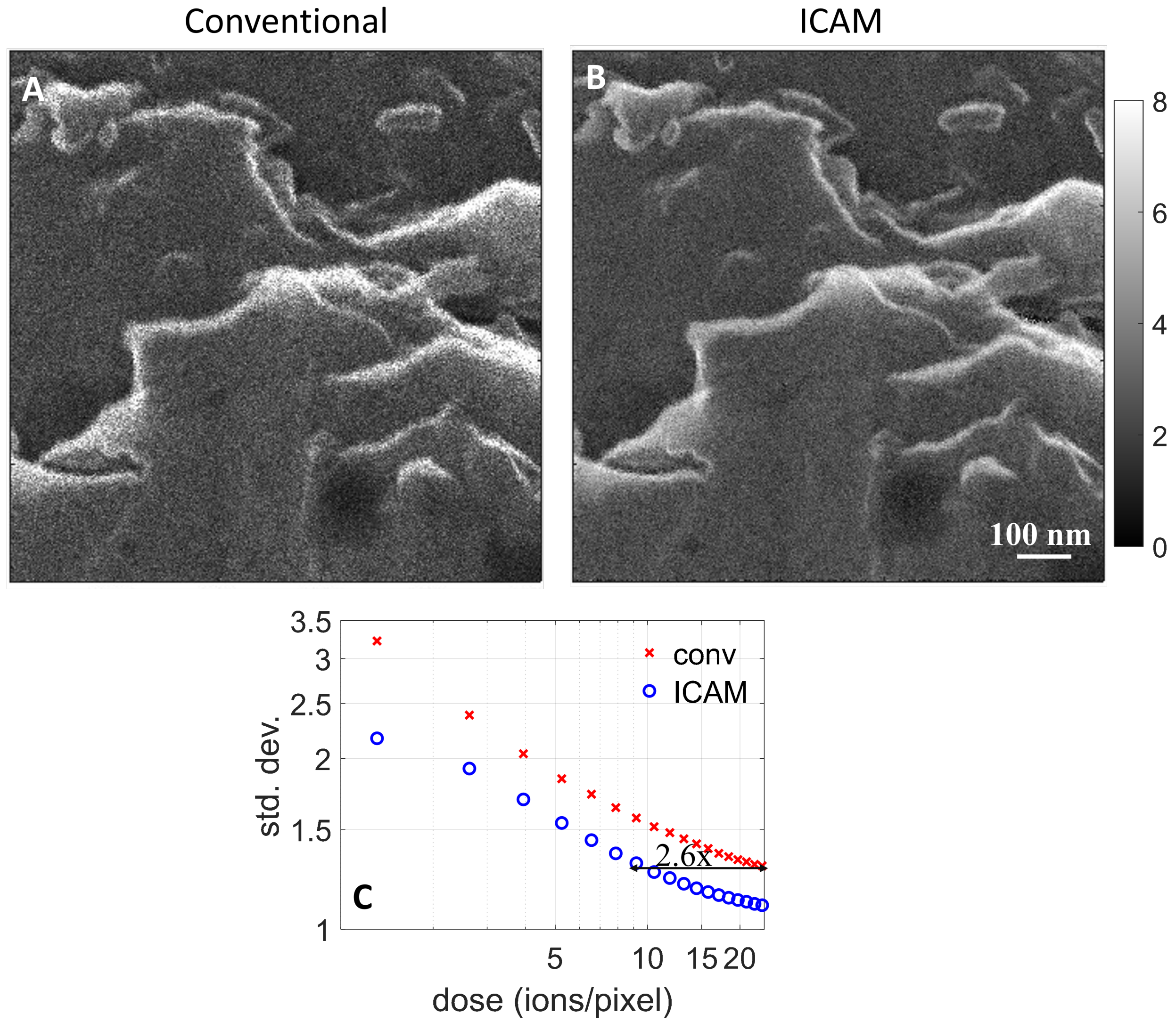

Figure S10: 1 μm field-of-view image of silicon sample at dose $\lambda = 22$ ions. (A) Conventional image. (B) ICAM image. (C) Comparison of image standard deviation vs dose for conventional and ICAM imaging.

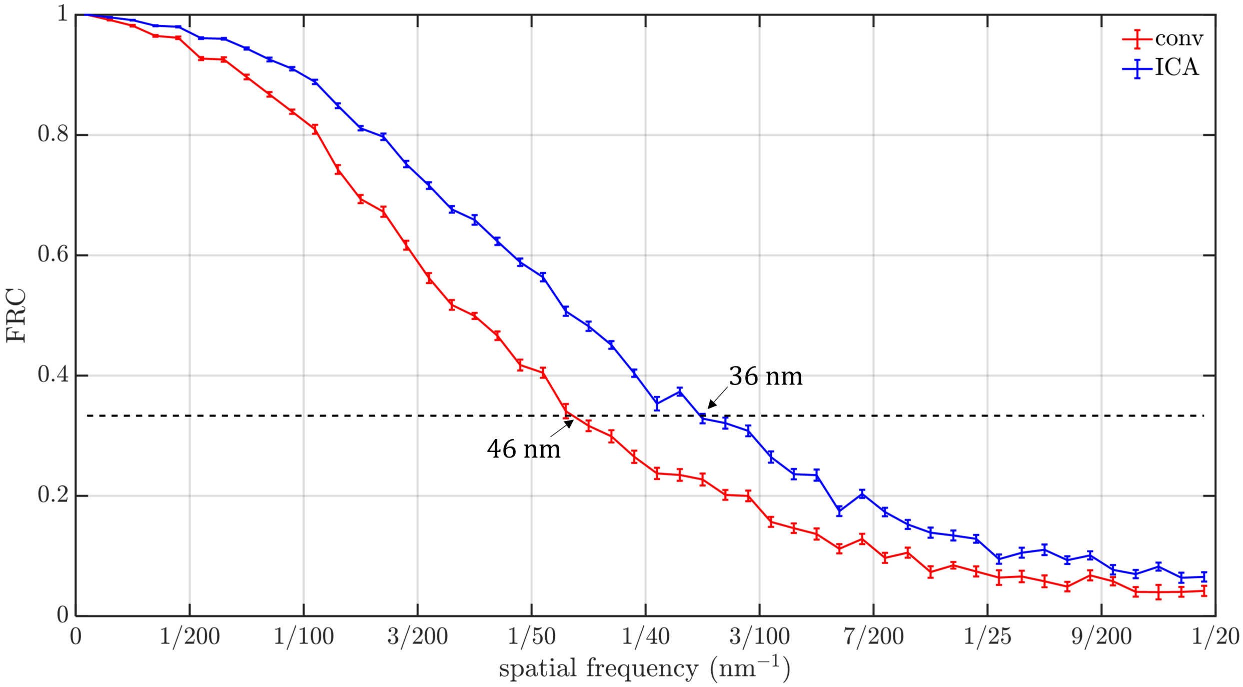

Figure S11: Fourier Ring Correlation for conventional (red) and ICA (blue) estimators. The 1/3 criterion resolution is $46\,\mathrm{nm}$ for conventional and $36\,\mathrm{nm}$ for ICAM.

The Fourier Ring Correlation (FRC) [7, 8, 9] is a resolution metric commonly used in cryo-electron microscopy [10]. This metric attempts to find the highest spatial frequency at which the Fourier spectrum of the image contains useful signal by looking at the normalized cross-correlation between the Fourier transforms of two half-dataset images, computed within rings of increasing spatial frequency. The resolution is defined as the inverse of the spatial frequency at which the FRC is greater than a pre-decided threshold. A number of different thresholds are used for defining resolution [10, 11]. Here, we will use 1/3 threshold. Similar to our calculation of Saxton's SNR metric, we repeated our calculation of the FRC for 1000 independent half-dataset divisions. Figure S11 shows the average FRC curves for the conventional (red curve) and ICA (blue curve) estimators, along with error bars that correspond to the standard deviation of each FRC value over the 1000 dataset divisions. The 1/3 resolution criterion is indicated by the horizontal black dashed line. The resolution is $46\,\mathrm{nm}$ for the conventional estimator and $36\,\mathrm{nm}$ for the ICA estimator.

Gold on silicon sample: In the paper, we characterized this sample by calculating Thong's SNR, as well as the dose reduction for the silicon and gold regions. We can further exploit the binary nature of this sample to define a distinguishability metric D between the bright and dark levels as

$$D = \frac{|\eta_1 - \eta_2|}{\sqrt{\sigma_1 \sigma_2}},$$

where η_1 and η_2 are the mean SE yields and σ_1 and σ_2 are the standard deviations of the SE yield values for the two pixel types. A higher value of this metric would imply greater ability to distingush between gold and silicon pixels, and consequently, higher image quality. We get $D = 1.87$ for the ICAM image and $D = 1.50$ for the conventional image.

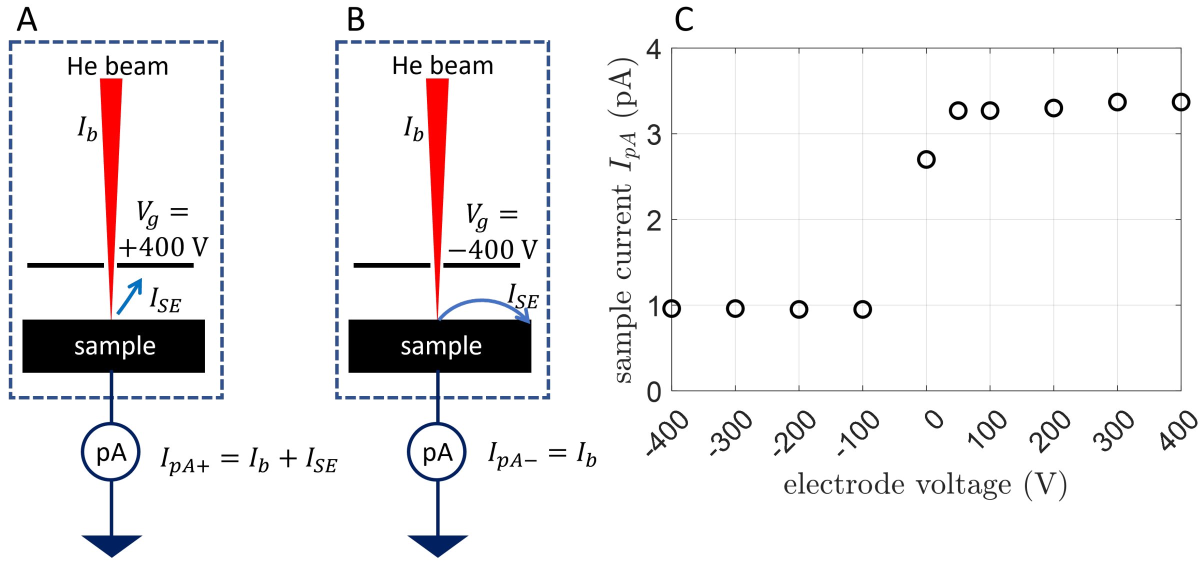

Figure S12: Measurement of bulk sample SE yield. (A) When electrode voltage V_g is highly positive, emitted SEs are drawn away from the sample. The total measured sample current is the sum of the beam and SE currents. (B) When V_g is highly negative, emitted SEs are repelled back towards the sample. The measured sample current is just the beam current. (C) Plot of sample current as a function of electrode voltage.

S8 Calculation of SED Detector Quantum Efficiency

As discussed briefly in the paper, our measured SE yield values are not absolute, since we did not separate the effect of non-ideal detective quantum efficiency (DQE) from the calculation. The DQE is defined as the fraction of emitted SEs that produce a signal on the SED [4, 5]. Since the DQE of the SED can depend on several factors, such as the relative placement of the sample and the SED, the working distance, and the type of detector (in-lens vs. in-chamber), we only quoted the as-measured SE yield values in the paper.

Nevertheless, the DQE for a particular experimental geometry can be measured. Our measurement of the SE yield through the methods described in the paper are equivalent to measuring the average number of detected SEs. If we can independently measure the average number of emitted SEs, we can calculate the DQE. For this purpose, we used the setups depicted in Figure S12, A and B. We placed an annular copper electrode above a featureless silicon sample, and we measured the sample current I_{pA} at various electrode voltages using a picoammeter. When the electrode voltage V_g is highly positive, as depicted in Figure S12A, we expect I_{pA} to be the sum of the beam current I_b and the SE current I_{SE}. When V_g is highly negative, SEs are repelled away from the electrode and back to the sample, and I_{pA} will be equal to I_b. Therefore, the SE yield is given by $\widehat{\eta} = (I_{pA+}/I_{pA-}) - 1$. Figure S12C is a plot of I_{pA} as a function of the applied electrode voltage, measured at $I_b = 1\,\text{pA}$. We can see that, as expected, when V_g is highly negative, $I_{pA} = I_b$, and when the grid voltage is highly positive, I_{pA} is higher than I_b. From these measurements, we extract $\widehat{\eta} = 2.51$. For this sample, the ICA estimator gave $\widehat{\eta} = 2.20$. From the ratio between these numbers, we get DQE = 88%.

S9 Beam Current Sensitivity of Conventional and ICA Estimators

From the expressions for the estimators, we can compute their sensitivities to changes in the incident current (equivalently, changes in dose λ). From eq. (S4), it is straightforward to conclude that

$$\frac{\partial \widehat{\eta}_{\text{conv}}}{\widehat{\eta}_{\text{conv}}} = -\frac{\partial \lambda}{\lambda}.$$

Therefore, 10% fluctuation in λ causes the the conventional SE yield estimate to fluctuate by 10%. For the ICA estimator, we can use eq. (S9) to write

$$\begin{aligned}\frac{\partial \widehat{\eta}_{\rm ICA}}{\widehat{\eta}_{\rm ICA}} &= -\frac{\partial \lambda}{\lambda}\left(\frac{e^{-\widehat{\eta}_{\rm ICA}}}{\widetilde{M}/\lambda - e^{-\widehat{\eta}_{\rm ICA}}(\widehat{\eta}_{\rm ICA}-1)}\right)\\ &\approx -\frac{\partial \lambda}{\lambda}\left(\frac{1}{e^{\widehat{\eta}_{\rm ICA}}-\widehat{\eta}_{\rm ICA}}\right).\end{aligned}$$

Here the first equality follows from differentiating eq. (S9) with respect to λ. We approximate $\widetilde{M}$ by its mean value of $\lambda(1-e^{-\eta})$ to get the final expression. At $\eta = 2.75$, a 10% fluctuation in λ causes the ICA SE yield estimate to fluctuate by only $\sim 0.8\%$.

S10 Relative Error Dependence on Scaling of SE Yield

The use of ICAM in SEI creates quantitative estimates of SE yield η, replacing the qualitative nature of most SEI. Nevertheless, there are possibilities for scaling of η that prompt us to comment on how the size of η affects imaging performance. For example, varying SED collector bias will change the effective η (see Section S3.6), and replacing helium with heavier ions at the same velocity will generally increase η [12]. We argue below that ICAM makes having large values of η favorable, whereas conventional imaging gives little to no benefit for large η.

For simplicity, let us assume noiseless SED ($c_\sigma = 0$). Then eq. (S5) gives

$$\mathrm{MSE}_{\rm conv} = \frac{\eta(\eta+1)}{\lambda}. \tag{S14}$$

As a scale-invariant accuracy metric, consider *relative error* defined by normalizing the root mean-squared error by the quantity of interest:

$$\mathrm{RE}_{\rm conv} = \frac{\sqrt{\mathrm{MSE}_{\rm conv}}}{\eta} = \frac{\sqrt{\eta(\eta+1)}}{\eta\sqrt{\lambda}} = \frac{\sqrt{1+1/\eta}}{\sqrt{\lambda}}. \tag{S15}$$

(Since the estimator is unbiased, this quantity may be familiar as the *coefficient of variation*.) For comparison, in the idealized case of noiseless SED, we can expect the performance of ICAM to follow predictions from Fisher information calculations [1]. These suggest

$$\mathrm{MSE}_{\rm ICA} = \frac{\eta}{\lambda(1-\eta e^{-\eta})}, \tag{S16}$$

which yields relative error

$$\mathrm{RE}_{\rm ICA} = \frac{\sqrt{\mathrm{MSE}_{\rm ICA}}}{\eta} = \frac{(1-\eta e^{-\eta})^{-1/2}}{\sqrt{\eta}\sqrt{\lambda}}. \tag{S17}$$

To compare eqs. (S15) and (S17), first note that the numerators satisfy

$$(1-\eta e^{-\eta})^{-1/2} < \sqrt{1+1/\eta} \qquad \text{for all } \eta > 0, \tag{S18}$$

with

$$\lim_{\eta\to\infty} (1-\eta e^{-\eta})^{-1/2} = 1 \qquad \text{and} \qquad \lim_{\eta\to\infty} \sqrt{1+1/\eta} = 1. \tag{S19}$$

Additionally, the denominator of eq. (S17) shows that $\mathrm{RE}_{\rm ICA}$ decays as $\eta^{-1/2}$ with increasing η. The relative errors are plotted in Figure S13 for $\lambda = 100$. Notice that the relative error for conventional image formation approaches a floor of $\lambda^{-1/2} = 0.1$ whereas the relative error for ICAM is always lower and exhibits the expected $\eta^{-1/2}$ decay (slope of $-1/2$ on a log-log plot).

This analysis shows that the computational methods for image formation generate significantly different behaviors: under conventional image formation, there is little to no benefit to increasing η, whereas ICAM produces images of better accuracy when η is increased. The improvement in relative error with increasing η creates a new trade-off between image quality and sample damage that could change the incident particle type and energy for optimal imaging resolution.

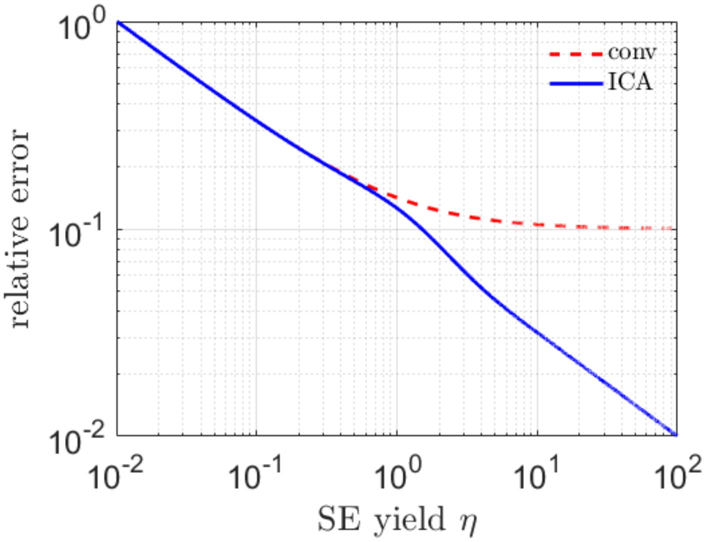

Figure S13: Comparison of relative errors for conventional and ICA estimation [see eqs. (S15) and (S17)].

Supplementary references

[1] M. Peng, J. Murray-Bruce, and V. K. Goyal, "Time-resolved focused ion beam microscopy: Modeling, estimation methods, and analyses," *IEEE Trans. Comput. Imaging*, vol. 7, pp. 547–561, 2021.

[2] J. A. Notte, S. Carousso, and B. W. Ward, "Ion beam imaging." World Intellectual Property Organization publication WO 2009/079195 Al, 25 June 2009.

[3] A. Agarwal, M. Peng, and V. K. Goyal, "Continuous-time modeling and analysis of particle beam metrology," *IEEE J. Sel. Areas Inform. Theory*, vol. 4, pp. 61–74, 2023.

[4] D. C. Joy, "Noise and its effects on the low-voltage SEM," in *Biological Low-Voltage Scanning Electron Microscopy* (H. Schatten and J. B. Pawley, eds.), pp. 129–144, New York, NY: Springer, 2008.

[5] A. Agarwal, J. Simonaitis, and K. K. Berggren, "Image-histogram-based secondary electron counting to evaluate detective quantum efficiency in SEM," *Ultramicroscopy*, vol. 224, p. 113238, 2021.

[6] W. O. Saxton, "Basic software for digital image handling - reprinted from advances in electronics and electron physics, supplement 10, 1978.," in *Computer Techniques for Image Processing in Electron Microscopy* (M. Hÿtch and P. W. Hawkes, eds.), vol. 214 of *Advances in Imaging and Electron Physics*, pp. 199–266, Amsterdam: Elsevier, 2020.

[7] M. van Heel, W. Keegstra, W. Schutter, and E. F. J. van Bruggen, "Arthropod hemocyanin structures studied by image analysis," *Life Chemistry Reports, Suppl. 1, "The Structure and Function of Invertebrate Respiratory Proteins"', EMBO workshop*, pp. 69–72, 1982.

[8] W. O. Saxton and W. Baumeister, "The correlation averaging of a regularly arranged bacterial cell envelope protein," *J. Microscopy*, vol. 127, pp. 127–138, 1982.

[9] M. van Heel, "Similarity measures between images," *Ultramicroscopy*, vol. 21, pp. 95–100, 1987.

[10] P. B. Rosenthal and R. Henderson, "Optimal determination of particle orientation, absolute hand, and contrast loss in single-particle electron cryomicroscopy," *J. Molecular Biology*, vol. 333, pp. 721–745, Oct. 2003.

[11] C. Sorzano, J. Vargas, J. Otón, V. Abrishami, J. de la Rosa-Trevín, J. Gómez-Blanco, J. Vilas, R. Marabini, and J. Carazo, "A review of resolution measures and related aspects in 3d electron microscopy," *Progress in Biophysics and Molecular Biology*, vol. 124, pp. 1–30, Mar. 2017.

[12] U. Fehn, "Variance of ion-electron coefficients with atomic number of impacting ions," *International Journal of Mass Spectrometry and Ion Physics*, vol. 21, no. 1, pp. 1–14, 1976.
